# The efficacy and safety of immunotherapy in patients with advanced NSCLC: a systematic review and meta-analysis

**DOI:** 10.1038/srep32020

**Published:** 2016-08-25

**Authors:** Liang Zhou, Xi-Ling Wang, Qing-Long Deng, Yan-Qiu Du, Nai-Qing Zhao

**Affiliations:** 1 Department of Biostatistics, School of Public Health and Key Laboratory of Public Health Safety, Fudan University, Shanghai 200032, People’s Republic of China; 2 Department of Preventive Medicine, Fudan University, Shanghai 200032, People’s Republic of China

## Abstract

Immunotherapy is a novel treatment for advanced non-small cell lung cancer (NSCLC) patients. Immunotherapy includes two main broad classes of therapeutic vaccines and immune checkpoint inhibitors, as well as cytokines, biological response modifiers and cellular therapy. The present systematic review and meta-analysis aims to evaluate the efficacy and safety of different classes of immunotherapy in patients with advanced NSCLC. Literature search was done on Medline, Embase and Cochrane Library. The primary endpoints were overall survival (OS) and grade ≥3 adverse events. Twenty randomized controlled trials were finally identified in our study. Efficacy analysis indicated an improvement of OS in advanced NSCLC patients after treating by therapeutic vaccines and immune checkpoint inhibitors, but not for other immunomodulators. Safety analysis showed that immunotherapy was well-tolerated. All kinds of grade ≥3 adverse events were similar between experimental group and control group except that neutropenia and thrombocytopenia had a higher incidence in patients received vaccines. In conclusion, immunotherapy is a promising treatment for advanced NSCLC patients. Our findings will be further confirmed and supplemented by several phase II and phase III RCTs which are going to complete in near future.

Lung cancer was the most frequently diagnosed cancer and the leading cause of cancer deaths in men according to the 2012 global cancer statistics^1^. Non-small cell lung cancer (NSCLC) is the major type of lung cancer, which accounts for approximately 85% of all cases^2^. However, the treatment and prognosis of NSCLC are far from satisfactory. About 75% NSCLC cases are diagnosed at an advanced stage with unresectable situation^3^, and 60‒70% patients who receive surgery finally exhibit postoperative recurrence and metastasis^4^. Platinum-doublet chemotherapy is the standard first-line treatment for patients with stage IIIB or stage IV NSCLC^5^, but patients usually suffer from limited efficacy and significant safety issues^6^. Novel treatments such as chemoradiotherapy and targeted therapies have been conducted in massive attempts over the last decade, but the 5-year survival of patients with NSCLC remains lower than 20%^7^.

In the past three years, more attention has been attracted to immunotherapy, which is likely to be a promising treatment for advanced NSCLC patients^8^,^9^,^10^,^11^,^12^,^13^,^14^,^15^,^16^. A randomized open-label controlled trial, which enrolled 272 patients, demonstrated a statistically significant improvement in overall survival (HR = 0.59, 95%CI: 0.44‒0.79) for patients randomized to nivolumab (a fully humanized antibody against PD-1) as compared with docetaxel at the prespecified interim analysis^17^. This study contributes to the quick approval of nivolumab to treat metastatic squamous NSCLC patients by FDA in 2015. The current forefront of immunotherapy for NCSLC involves two broad classes of agents, namely allogeneic vaccines and immune checkpoint inhibitors^18^. Therapeutic cancer vaccines are designed to stimulate immune cells to target specific tumor-associated antigens, while immune checkpoint inhibitors can expand T-cells response and enhance anti-tumor effects through interfering with immune system’s auto-regulatory mechanisms^19^. Besides, clinical trials on cellular therapies, cytokines and biological response modifiers are also reported. However, findings regarding the efficacy and safety of immunotherapy are not always consistent. Oral talactoferrin performed well in two phase II trials, but it ultimately failed to improve overall survival in the treatment group compared to placebo group for patients with advanced NSCLC in the phase III FORTIS-M trial^20^,^21^,^22^. Meta-analysis is an approach to assess the overall efficacy and safety of immunotherapy by pooling patient samples. A previous meta-analysis based on 12 randomized controlled trails has revealed the benefit of immunotherapy on overall survival (HR = 0.95, 95%CI 0.92‒0.98) with handful adverse events^6^. However, the study may not be accurate in classifying immunotherapy treatments. They included 3 trials of cetuximab and 1 trial of trastuzumab as monoclonal antibodies subgroup, which should in fact be classified as target therapy as their targets (EGFR and HER-2 respectively) were related to the growth of tumors’ blood vessels instead of human’s immune system. Therefore, our systematic review and meta-analysis aims to provide more reliable and up-to-date evidence on the efficacy and safety of immunotherapy for advanced NSCLC patients.

## Method

### Study selection criteria

Randomized controlled trials (RCTs) involving patients with histologically confirmed unresectable NSCLC (stages IIIA, IIIB and IV) or metastatic disease were eligible. The treatment of experimental group should be either immunotherapy combined chemotherapy or immunotherapy as monotherapy, and the intervention of control group should be anyone of chemotherapy, placebo or best support care. Publications and unpublished trials in English language from 2003 to current were searched in our study.

### Search strategies

Searches were done on 13 April 2015 and were updated on 13 March 2016. Studies with keywords in MeSH terms “carcinoma, non-small-cell lung” and “immunotherapy” were retrieved from MEDLINE and EMBASE. Searching filters were applied to guarantee the relevance of results (appendix). The same search strategies were applied in Cochrane Library to gather unpublished trials and abstracts of the American Society of Clinical Oncology (ASCO) annual meeting since 2011 to 2015 were screened to identify relevant studies.

### Data extraction

Literature screening and data extraction were carried out by two independent reviewers and then verified by the third reviewer. The reviewers had to fill in a pre-defined form to collect data for included studies and state reasons for excluded studies. Efficacy analysis was based on intention-to-treat population. The primary endpoint was overall survival (OS), which was defined as the time from randomization to either death or censor. To evaluate safety, number of patients that presented adverse events (grade ≥3) were collected. Adverse events (grade ≥3) reported by more than 2 trials within the same immunotherapy treatments were analyzed. Hematological events included neutropenia, leukopenia, anemia and thrombocytopenia, and non-hematological adverse events included dyspnea, diarrhea, asthenia or fatigue, nausea or vomiting.

In addition, we assessed the validity of methodology for included studies. Data were sought on patients’ inclusion criteria and baseline characteristics, treatment allocation, randomization method, blinding, loss to follow-up and treatment completion. We used Jadad scale to assess the quality of trials^23^ with scores less than 3 indicating high risk of bias.

### Statistical analysis

For each included study, hazard ratio (HR) and its 95% confidence interval (CI) were collected for survival data. If HR was not available in publications, the point estimate of HR was calculated by median OS in each group and its logarithmic standard error was estimated either by numbers of death events in each group or by *p* value of log-rank test. If HR can neither be collected directly nor calculated, survival curve plots were extracted by Engauge Digitizer software and then transformed by specialized form^24^,^25^. For dichotomous data of adverse events (AEs), risk ratio (RR) was calculated by number of events and number of patients at risk in each group. An HR less than 1 favored the efficacy of immunotherapy and RR less than 1 presented the good tolerance of immunotherapy.

Stratification analyses were conducted for the following groups: immunotherapy combined chemotherapy versus chemotherapy with or without placebo (I + C vs. C + (P)), single immunotherapy versus placebo (I vs. P), or immunotherapy versus chemotherapy (I vs. C). To be more conservative, pooled HR and its 95% CI were estimated by using a random-effect model even if heterogeneity was not detected among studies^26^. Sensitivity analysis was done to test the stability of pooled results by excluding trials with high risk of bias. All analyses were performed by Stata 11.

## Results

**Figure 1:**
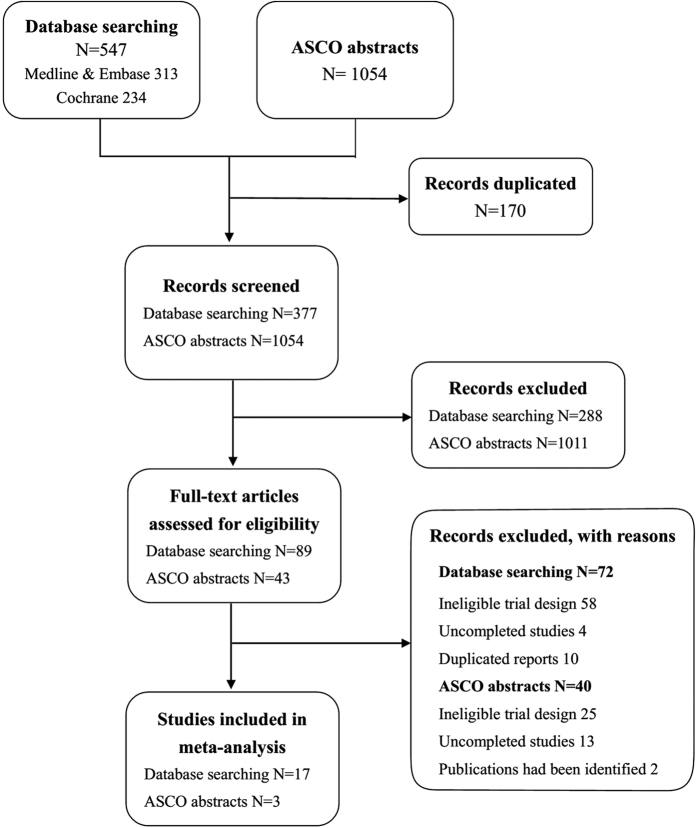
Study flow diagram.

We initially identified a total of 547 papers from database search and 1054 ASCO abstracts. 170 papers were excluded due to duplication. 89 papers and 43 ASCO abstracts fulfilled our inclusion criteria after reading the titles and abstracts. The papers were further assessed for eligibility by reading the full-texts. Information of 43 ASCO abstracts were collected by their clinical trial numbers through Clinicaltrials.gov or Google Scholar. Finally, we included 20 clinical trials^17^,^20^,^21^,^22^,^27^,^28^,^29^,^30^,^31^,^32^,^33^,^34^,^35^,^36^,^37^,^38^,^39^,^40^,^41^,^42^, 17 from database search and 3^31^,^35^,^38^ from ASCO abstracts (Fig. 1).

The characteristics of included 20 trials were listed in Table 1. Four trials^32^,^34^,^39^,^42^ involved patients with stage IIIA NSCLC, but were restricted to unresectable NSCLC patients. Survival data were extracted from all eligible trials. HRs of overall survival (OS) were reported directly in sixteen trials, but were estimated by median survival time of treatment group and control group for three trials^27^,^40^,^42^. The remaining one trial only provided survival curve^41^, so HR was calculated through curve data.

### Therapeutic Vaccines

Etiologically, the infiltration of Treg cells leaded to the immune evasion of tumor cells, which explained the weak immunogenicity of NSCLC^43^. Tumor vaccines were designed to prompt an immune response to tumor-associated antigens through active immunization with either whole-cell or antigen-specific vaccines^44^.

Belagenpumatucel-L was an allogeneic tumor cell vaccine that were transfected with a TGF-β2 antisense plasmid. Giaccone *et al*.’s study^34^ found no differences on overall survival between belagenpumatucel-L and placebo in the ITT analysis. But a prespecified COX regression analysis suggested that early enrollment after first-line therapy and prior chemoradiation were positive prognostic factors that favored belagenpumatucel-L. In this trial, injection site reaction, induration and erythema were more frequent in belagenpumatucel-L group, but they were all classified as grade 1 or 2 AEs. These safety results were consistent with previous study^45^ and indicated the well-tolerance of belagenpumatucel-L.

Tecemotide (L-BLP25) and TG4010 were antigen-specific vaccines designed for inducing a T-cell response to aberrant MUC1 protein. Several RCTs have reported that the vaccines improved overall survival compared with control group^29^,^30^,^32^,^35^, but neither of them reached significant level in the ITT analyses. Subgroup analyses stratified by patients’ biological status (pretreatment or histopathology) provided information for the patients’ screening for immunotherapy. Safety results were considerable that grade 3 or 4 AEs or SAEs were similar between experimental group and control group. Pneumonia and dyspnea were slightly more common in patients with L-BLP25 than placebo^32^. In patients with a high percentage of CD16 + CD56 + CD69 + lymphocytes at baseline, there was a significantly higher incidence of serious adverse events in the TG4010 group (15 of 21) than in the chemotherapy alone group (5 of 16)^30^.

Epidermal Growth Factor (EGF) promoted tumor cell proliferation and survival upon binding to its receptor^46^. The EGF vaccine (CIMAVax) can simulate an antibody-mediated immune response against EGF ligand. A phase II randomized controlled trial^27^ involving 80 patients demonstrated a remarkable improvement in survival for patients with substantial immunological response when compared with BSC group (11.7 months vs. 5.33 months, *p* = 0.002). But in the ITT population, the difference was not significant which may result from the small sample size. The vaccine was very well tolerated that no grade 3 or 4 AEs or SAEs were attributed to the study drug among three trials^27^,^46^,^47^. The most common AEs included chills, fever, injection-sit pain, nausea and vomiting.

**Figure 2:**
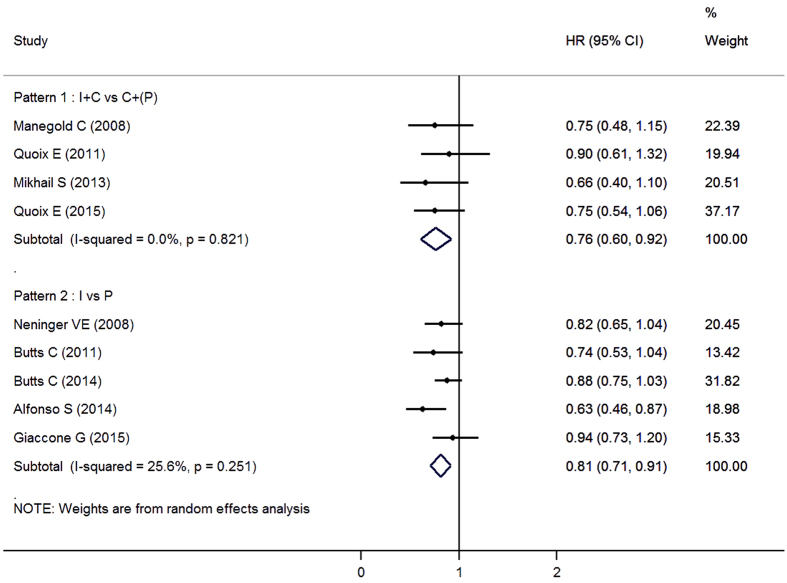
Forest plot of overall survival in advanced NSCLC patients who received therapeutic vaccines with or without chemotherapy compared to control therapies. I: immunotherapy; C: chemotherapy; P: placebo.

There were also RCTs exploring other types of vaccines for advanced NSCLC. Racotumomab-alum was an anti-idiotype vaccine mimicking the NeuGcGM3 tumor-associated ganglioside^33^. Bavituximab was a novel monoclonal antibody that targetd phosphatidylserine (PS), binding PS to simulate an immune response^31^. Both of the drugs were reported to be safe and well-tolerated. PF-3512676 was a synthetic TLR9-activating oligo deoxy nucleotide that mimic the natural ligand of TLR9, thereby inducing a cascade of immune reactions and potentially promoting an antitumor immune response^28^. However, Manegold *et al*.’s study suggested that grade 3 or 4 hematological AEs were more frequent in the PF-3512676 plus chemotherapy arm than chemotherapy-alone arm. But these issues did not result in any clinically significant sequelae. Efficacy results of these trials were showed in Fig. 2.

In pooled analyses, therapeutic vaccines significantly improved survival as compared with placebo, with an HR of 0.81 (95%CI, 0.71 to 0.91). Results were similar for chemotherapy combined vaccines versus chemotherapy alone (Fig. 2; HR, 0.76; 95%CI, 0.60 to 0.92). Neutropenia and thrombocytopenia were more common in the experimental group, while other types of AEs were similar between experimental group and control group (Table 2). There were no differences in incidence of grade ≥3 AEs or SAEs between two groups.

### Immune Checkpoint Inhibitors

Cytotoxic T-lymphocyte-associated protein 4 (CTLA-4) was a molecular that had the same ligands as CD28. It inhibited the activation of cytotoxic T-cells by interfering the action of CD28 after antigen presentation^48^. Ipilimumab was a monoclonal antibody targeting CTLA-4, thus enhanced the T cells response. Lynch *et al*.’s study^36^ demonstrated a trend of survival improvement among phased ipilimumab arm (two doses of placebo plus paclitaxel and carboplatin followed by four doses of ipilimumab plus paclitaxel and carboplatin) when compared with control arm (six cycles of paclitaxel, carboplatin and placebo). Subgroup analyses indicated that phased ipilimumab appeared to show improved efficacy for squamous histology (HR, 0.48; 95%CI, 0.22 to 1.03). Grade 3 rash, diarrhea and colitis were noted in phased ipilimumab arm or concurrent ipilimumab arm. The incidence of grade 3 or 4 immune-related adverse events was higher in patients receiving ipilimumab (15%, 20%, and 6% for phased ipilimumab, concurrent ipilimumab, and the control, respectively).

**Figure 3:**
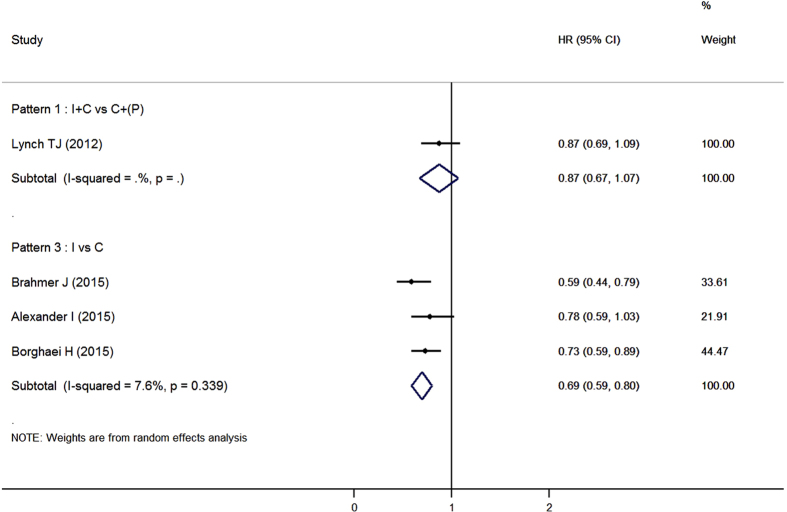
Forest plot of overall survival in advanced NSCLC patients who received immune checkpoint inhibitors with or without chemotherapy compared to control therapies. I: immunotherapy; C: chemotherapy; P: placebo.

Programmed cell death 1(PD-1) was a co-inhibitory molecule receptor expressed by activated T cells, and its ligands PD-L1 was expressed on tumor cells as well as stromal cells. PD-1/PD-L1 pathway played an important role in immune-mediated tolerance of NSCLC^49^. Nivolumab (also known as BMS-936558) was a human IgG4 anti-PD-1 antibody. It can enhance the cytotoxic activity of T lymphocytes by blocking the ligand activation of PD-1. Two phase III trials^17^,^37^ had demonstrated a remarkable efficacy of nivolumab as compared with docetaxel in patients with advanced squamous or non-squamous NSCLC (Fig. 3). Fatigue, decreased appetite and asthenia were the most frequently reported treatment-related AEs in both studies. Nivolumab had a better tolerance than docetaxel. In Brahmer *et al*.’s study, grade 3 or 4 treatment-related AEs occurred in 7% of patients received nivolumab and in 55% of patients received docetaxel. Similarly, 10% and 54% of patients experienced grade 3 or 4 treatment-related AEs in Borghaei *et al*.’s study respectively. Drug-related adverse events of special interest such as hypothyroidism and pneumonitis were observed in both trials. As mentioned in a prior phase I study, these adverse events were regarded to have potential immune-related causes^50^. Notably, the correlation between PD-L1 expression and survival were inconsistent within two studies, which may due to the different histopathology of NSCLC^37^.

MPDL3280A was another engineered monoclonal antibody of IgG1 isotype against PD-1. Results of an interim analysis of a randomized phase II study of MPDL3280A compared with docetaxel in patients with locally advanced or metastatic NSCLC had been presented at ASCO annual meeting in 2015^38^. In this study, improved efficacy of MPDL3280A was observed among patients with increasing PD-L1 expression (HR, 0.63; 95%CI, 0.42–0.95), but not for patients with the lowest PD-L1 levels (HR, 1.22; 95%CI, 0.69–2.14). This agent was more tolerable than docetaxel in this trial. 43% of patients in MPDL3280A arm and 54% of patients in docetaxel arm experienced grade ≥3 AEs.

In pooled analyses, anti-PD-1 antibodies achieved inspiring improvement on survival (HR, 0.69; 95%CI, 0.59 to 0.80) in patients with advanced NSCLC when compared with docetaxel (Fig. 3). Immune checkpoint inhibitors were well-tolerated. Grade 3 or 4 hematological adverse events were less frequent in experimental group while there were no differences among diarrhea, nausea and vomiting (Table 2).

### Other Immunomodulators and Cellular Therapy

Talactoferrin was an orally immunomodulatory protein that interacted with gut-associated lymphoid tissue, prompting the maturation of dendritic cells and thus simulated a strong anti-tumor immune response^20^. Two randomized phase II study had showed a promising efficacy of talactoferrin in advanced NSCLC patients^20^,^21^. But in the phase III FORTIS-M trial, no differences on overall survival were identified between talactoferrin and placebo^22^. Ramalingam *et al*. pointed out that the variety of patients’ pretreatment and population could potentially have impacted the inconsistent outcomes. The safety and tolerability of talactoferrin were verified by these trials. No drug-related SAEs were reported.

Interleukin 2 (IL-2) was a type of cytokine that regulated the activities of lymphocytes. Tumor-induced immunosuppressive phenomena were reversible *in vitro* by the addition of exogenous IL-2^40^. A phase III randomized multicenter trial^40^ comparing chemotherapy with or without low dose IL-2 in patients with advanced NSCLC failed to demonstrate any survival benefits of IL-2. In this study, more patients experienced grade 4 AEs in chemotherapy plus IL-2 group than chemotherapy alone group (50 vs. 27).

SRL172 was a suspension of killed Mycobacterium vaccae. It can activate antigen-presenting cells and natural killer cells as well as suppress the activation of Treg cells. O’Brien *et al*.’s study^39^ found no statistical difference in overall survival between the chemotherapy plus SRL172 group and the chemotherapy alone group. But quality of life was higher in patients received chemotherapy plus SRL172. Treatment-related SAEs were more frequent in the chemotherapy plus SRL172 group (106/210 patients) than the chemotherapy alone group (80/209 patients).

**Figure 4:**
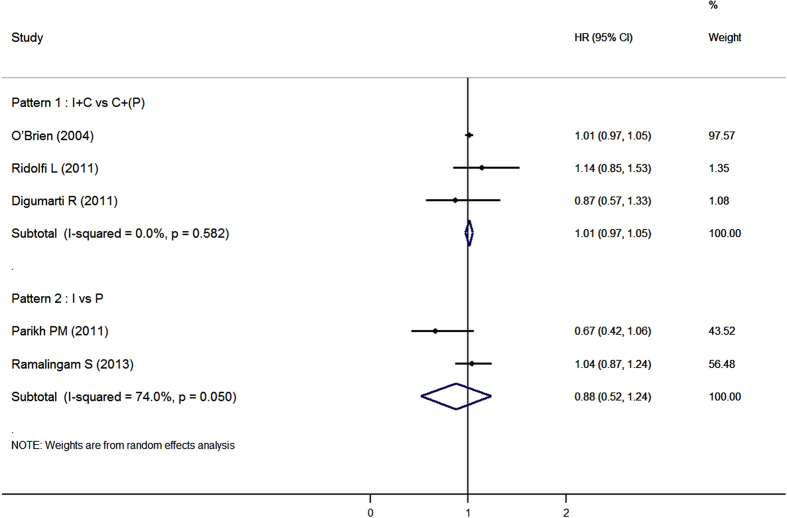
Forest plot of overall survival in advanced NSCLC patients who received other immunomodulators with or without chemotherapy compared to control therapies. I: immunotherapy; C: chemotherapy; P: placebo.

Pooled analysis suggested that there were no significant improvements in overall survival for these immunomodulators, either combined with chemotherapy or used as monotherapy (Fig. 4). In general, Episodes of grade ≥3 AEs were similar between experimental groups and control groups except that thrombocytopenia were more common in patients received immunomodulators (Table 2).

Cytokine-induced killer (CIK) cells were a group of immune effector cells that can recognize malignant cells in the absence of major histocompatibility complex (MHC), allowing for a fast and unbiased immune reaction. Dendritic cells co-cultured with CIK cells (DC-CIK) can cause changes in the surface molecule expression of both population, thus leading to an improved cytotoxic activity^51^. Zhong *et al*.’s study^41^ demonstrated a trend of improved overall survival in chemotherapy plus DC-CIK group than chemotherapy alone group (HR≈0.87; log-rank *p* value = 0.18), while Wu *et al*.^42^ had indicated that the addition of CIK cells to chemotherapy statistically prolonged patients’ survival (median OS, 15 months versus 11 months; log-rank *p* value = 0.029). Non-infectious fever was mentioned in these two trials to be more frequent in the experimental groups. No treatment-related SAEs were reported.

### Sensitivity analysis

To assess the robustness of our results, sensitivity analysis was conducted to evaluate the influence of uncertain factors. When trials with high risk of bias were excluded in pooled analysis, the survival benefits of therapeutic vaccines and immune checkpoint inhibitors were still detected (Table 3).

Two phase III study of PF-3512676 (NCT00254904 and NCT00254891) had been terminated because of safety issues potentially related to the investigational drug. When Manegold *et al*.’s study was excluded, the differences of incidences of AEs tended to be non-significant.

## Discussion

In our meta-analysis, we demonstrated significant improvements on overall survival of therapeutic vaccines and immune checkpoint inhibitors in patients with advanced NSCLC, but the efficacy was not observed for other immunomodulators. Pooled analysis was not conducted for cellular therapy because of small number of related trials. Multicenter studies in different population were needed to assess the efficacy and safety of cellular therapy in advanced NSCLC patients.

Our findings suggest that immunotherapy agents, which could simulate a specific anti-tumor response, seems to be more powerful than those mainly enhance the whole immune response level. Besides, the expression of immunotherapy targets might significantly relate to survival, which indicates that patients’ screening for immunotherapy should be taken into account in future study designs and clinical practice. Anti-PD-1 shows a very promising potential in the treatment of advanced NSCLC patients. Although the correlation between the expression of PD-L1 and patients’ survival are not consistent among trials, Borghaei *et al*. suggests to use nivolumab regardless of this issue for its better tolerance than docetaxel^37^.

In general, immunotherapy was relatively safe, especially for those simulating a specific anti-tumor reaction^52^,^53^. Although injection site reaction, fatigue, nausea and flu-like symptoms were common adverse events of immunotherapy, these AEs were moderate. Potential immune-related AEs (e.g. pneumonia colitis) were mentioned in several studies, while no severe immune-related AEs or autoimmune disease were reported.

Our findings of therapeutic vaccines are consistent with Min Wang’s meta-analysis^54^. But our study has an advantage over his study that we stratified our efficacy analysis by different control interventions. Trials that use placebo as control mostly involves patients who has received first-line treatment, while chemotherapy is often used for patients who are untreated before or have received less than 2 chemotherapy regimens (Table 1). Moreover, we include results from recent RCTs in our systematic review and meta-analysis. Besides, we consider SRL172 as another immunomodulators instead of vaccines, for its absence of specific targets. With respect to cellular therapy, Shuai Wang’s study^55^ identifies six clinical trials to assess the efficacy and safety of DC-CIK therapy in patients with NSCLC. However, we did not include five of the six studies in our analysis. The reasons are that two^56^,^57^ trials enrolled patients after surgery causing heterogeneity problems in pooling with advanced NSCLC patients, one trial^58^ used target therapy (erlotinib) instead of chemotherapy, one trial^59^ didn’t provide information on overall survival and other one trial^60^ was paired study.

Our meta-analysis has the limitation that it collects data from articles without individual patient data, so the impact of patients’ baseline characteristics such as age, race, stage and treatment regimens are unable to be explored.

In conclusion, therapeutic vaccines and immune checkpoint inhibitors improve overall survival with a well tolerance in advanced NSCLC patients. Agents simulating specific anti-tumor immune response seems to have better efficacy than other immunomodulators. Immunotherapy becomes a promising treatment for advanced NSCLC patients. Furthermore, RCTs investigate the efficacy and safety of dual immunotherapy, as well as combination of target therapy and immunotherapy, are ongoing^49^.

## Additional Information

**How to cite this article**: Zhou, L. *et al*. The efficacy and safety of immunotherapy in patients with advanced NSCLC: a systematic review and meta-analysis. *Sci. Rep.* **6**, 32020; doi: 10.1038/srep32020 (2016).

## Supplementary Material

Supplementary Information

Dataset 1

## Figures and Tables

**Table 1:** Characteristics of included trials.

Study	N	Stage	Previous treatment	Interventions	Jadad Score		
					Randomization	Blinding	Follow-up
**Therapeutic vaccines**							
Neninger VE 2008	80	IIIB–IV	Four to six cycles of platinum-based chemotherapy	EGF vaccine vs. BSC	2	0	1
Manegold C 2008	112	IIIB–IV	Chemotherapy-naive	Chemotherapy + PF-3512676 vs. chemotherapy	1	0	1
Butts, C 2011	171	IIIB–IV	Stable disease or an objective clinical response after first-line treatment	BLP-25 + BSC vs. BSC	1	0	1
Quoix E 2011	148	IIIB–IV	Absence of systemic therapy	TG4010 + GC vs. GC	2	0	1
Mikhail S 2013	121	IIIB–IV	One chemotherapy regimen	Bavituximab + D vs. P + D	1	1	0
Butts C 2014	1239	IIIA–IIIB	≥2 chemotherapy regimens	BLP-25 vs. P	2	1	1
Alfonso S 2014	176	IIIB–IV	Achieved CR, PR, or SD after the standard first-line therapy	Racotumomab vs. P	1	1	1
Giaccone G 2015	532	IIIA–IV	Stable disease or response following first-line treatment	Belagenpumatucel-L vs. P	2	1	1
Quoix E 2015	222	IV	Untreated before	TG4010 + CP vs. P + CP	1	1	0
**Immune checkpoint inhibitors**							
Lynch TJ 2012	204	IIIB–IV	Absence of systemic therapy	Ipilimumab + CP vs. P + CP	1	1	1
Brahmer J 2015	272	IIIB–IV	Recurrence after one prior platinum-containing regimen	Nivolumab vs. D	2	0	1
Borghaei H 2015	582	IIIB–IV	Recurrence after resection or progression after one prior platinum-based chemotherapy	Nivolumab vs. D	2	0	1
Alexander I 2015	287	IIIB–IV	Recurrence or progression after one prior platinum-containing regimen	MPDL3280A vs. D	1	0	0
**Other immunomodulators and cellular therapy**							
O’Brien 2004	419	IIIA–IV	Chemotherapy-naive	MVP + SRL172 vs. MVP	2	0	1
Digumarti R 2011	110	IIIB–IV	Absence of systemic therapy	TLF + CP vs. CP + P	1	1	1
Ridolfi L 2011	239	IIIB–IV	Untreated before	IL-2 + GC vs. GC	1	0	1
Parikh PM 2011	100	IIIB–IV	≥1 chemotherapy regimens	TLF + BSC vs. BSC + P	2	1	1
Ramalingam S 2013	742	IIIB–IV	≥2 chemotherapy regimens	TLF + BSC vs. BSC + P	2	1	1
Zhong Runbo 2011	28	IIIB–IV	Untreated before	DC/CIK + NP vs. NP	1	0	1
Wu Changping 2008	59	IIIA–IV	Taxanes naive	CIK + TP vs. TP	1	0	1

NP: vinorelbine with platinum chemotherapy; TP: docetaxel 75 mg/m^2^, day 1; cisplatin, 25 mg/m^2^, days 1–4, tri-weekly; CP: carboplatin plus paclitaxel;

GC: cisplatin plus gemcitabine; D: docetaxel; MVP: mitomycin, vinblastine and cisplatin or carboplatin; TLF: talactoferrin; P: placebo.

**Table 2:** Comparative adverse events (grade ≥3) of experimental group versus control group.

Adverse events	Number of trials	EXP group events/pts	CON group events/pts	Pooled RR (95%CI)
**Therapeutic vaccines**				
Neutropenia	4	133/298	93/295	1.25(1.01–1.55)
Leukopenia	2	39/115	20/116	1.23(0.55–2.78)
Anemia	4	44/298	33/295	1.44(0.65–3.15)
Thrombocytopenia	3	43/258	18/216	1.91(1.02–3.59)
Dyspnea	5	62/1333	39/824	0.97 (0.65–1.45)
Asthenia/Fatigue	6	53/1408	48/861	0.99 (0.69–1.44)
Nausea/Vomiting	2	7/148	7/109	0.69 (0.20–2.41)
Any grade ≥ 3 AEs	3	401/1150	247/645	0.95 (0.79–1.10)
Any serious AEs	7	507/1691	355/1169	0.94 (0.81–1.08)
**Immune checkpoint inhibitors**				
Neutropenia	3	2/485	121/462	0.03 (0.00–0.49)
Leukopenia	2	1/418	27/397	0.07 (0.01–0.83)
Anemia	3	7/485	17/462	0.34(0.07–1.72)
Diarrhea	3	7/485	9/462	0.81 (0.25–2.64)
Asthenia/Fatigue	3	12/485	41/462	0.27 (0.06–1.24)
Nausea/Vomiting	2	6/354	6/333	0.96 (0.32–2.90)
Any grade ≥3 AEs	3	93/485	255/462	0.31 (0.08–0.54)
**Other immunomodulators**				
Leukopenia	3	27/219	26/219	1.02(0.62–1.66)
Anemia	3	11/599	13/350	0.62(0.28–1.39)
Thrombocytopenia	2	69/172	37/166	1.75(1.20–2.55)
Dyspnea	3	57/599	53/350	0.67 (0.47–0.96)
Diarrhea	2	4/172	6/166	0.80 (0.01–81.54)
Asthenia/Fatigue	3	23/599	22/350	0.50 (0.17–1.48)
Nausea/Vomiting	3	22/219	26/219	0.85 (0.51–1.41)
Any grade ≥3 AEs	3	355/599	291/350	0.78 (0.51–1.05)

EXP: experimental; CON: control; pts: patients.

**Table 3:** Sensitivity analyses of efficacy of immunotherapy in patients with advanced NSCLC.

Type of immunotherapy*	Interventions	Number of trials	Pooled HR (95%CI)
Therapeutic vaccines	I vs. P	4	0.82 (0.70–0.94)
Immune checkpoint inhibitors	I vs. C	2	0.67 (0.53–0.80)
Other immunomodulators	I + C vs. C + (P)	2	1.01 (0.97–1.05)
	I vs. P	2	0.88 (0.52–1.24)

^*^Studies that Jadad score less than 3 were excluded. I: immunotherapy; P: placebo; C: chemotherapy.

## References

[b1] Torre L. A. . Global cancer statistics, 2012. CA: Cancer J Clin 65, 87–108 (2015). 25651787 10.3322/caac.21262

[b2] Madureira P., de Mello R. A., de Vasconcelos A. & Zhang Y. Immunotherapy for lung cancer: for whom the bell tolls? Tumor Biol 36, 1411–1422 (2015). 10.1007/s13277-015-3285-6 25736929

[b3] de Mello R. A. . EGFR exon mutation distribution and outcome in non-small-cell lung cancer: a Portuguese retrospective study. Tumour Biol 33, 2061–2068 (2012). 22843317 10.1007/s13277-012-0465-5

[b4] Zhao M., Li H., Li L. & Zhang Y. Effects of a gemcitabine plus platinum regimen combined with a dendritic cell-cytokine induced killer immunotherapy on recurrence and survival rate of non-small cell lung cancer patients. Exp Ther Med 7, 1403–1407 (2014). 24940447 10.3892/etm.2014.1574 PMC3991503

[b5] Wakelee H., Kelly K. & Edelman M. J. 50 Years of progress in the systemic therapy of non-small cell lung cancer. Am Soc Clin Oncol Educ Book 177–189 (2014). 24857075 10.14694/EdBook_AM.2014.34.177 PMC5600272

[b6] Wang J. . Strengths and weaknesses of immunotherapy for advanced non-small-cell lung cancer: a meta-analysis of 12 randomized controlled trials. PLoS One 7, e32695 (2012). 22403699 10.1371/journal.pone.0032695 PMC3293858

[b7] McCarthy F., Roshani R., Steele J. & Hagemann T. Current clinical immunotherapy targets in advanced nonsmall cell lung cancer (NSCLC). J Leukocyte Biol 94, 1201 (2013). 23695306 10.1189/jlb.0313121

[b8] Brahmer J. R. . KEYNOTE-024: Phase III trial of pembrolizumab (MK-3475) vs. platinum-based chemotherapy as first-line therapy for patients with metastatic non-small cell lung cancer (NSCLC) that expresses programmed cell death ligand 1 (PD-L1). J Clin Oncol 33, (suppl; abstr 8103) (2015).

[b9] Planchard D. . A phase III study of MEDI4736 (M), an anti-PD-L1 antibody, in monotherapy or in combination with Tremelimumab (T), versus standard of care (SOC) in patients (pts) with advanced non-small cell lung cancer (NSCLC) who have received at least two prior systemic treatment regimens (ARCTIC). J Clin Oncol 33, (suppl; abstr 8104) (2015).

[b10] Mok T. . Phase 3 KEYNOTE-042 trial of pembrolizumab (MK-3475) versus platinum doublet chemotherapy in treatment-naive patients (pts) with PD-L1-positive advanced non-small cell lung cancer (NSCLC). J Clin Oncol 33, (suppl; abstr 8105) (2015).

[b11] Gulley J. L. . Avelumab (MSB0010718C), an anti-PD-L1 antibody, in advanced NSCLC patients: A phase 1b, open-label expansion trial in patients progressing after platinum-based chemotherapy. J Clin Oncol 33, (suppl; abstr 8034) (2015).

[b12] Nishio M. . Phase II studies of nivolumab (anti-PD-1, BMS-936558, ONO-4538) in patients with advanced squamous (sq) or nonsquamous (non-sq) non-small cell lung cancer (NSCLC). J Clin Oncol 33, (suppl; abstr 8027) (2015).

[b13] Carbone D. P. . A phase III, randomized, open-label trial of nivolumab (anti-PD-1; BMS-936558, ONO-4538) versus investigator’s choice chemotherapy (ICC) as first-line therapy for stage IV or recurrent PD-L1+ non-small cell lung cancer (NSCLC). J Clin Oncol 32, (suppl; abstr 8128) (2014).

[b14] Bazhenova L. . An international, multicenter, randomized, double-blind phase III study of maintenance belagenpumatucel-l in non-small cell lung cancer (NSCLC): Updated analysis of patients enrolled within 12 weeks of completion of chemotherapy. J Clin Oncol 32, (suppl; abstr 8056) (2014).

[b15] Govindan R., Morris J. C., Rossi G. R., Vahanian N. N. & Link C. J. NLG-0301: An open-label, randomized phase 2B active control study of second-line tergenpumatucel-L immunotherapy versus docetaxel in patients with progressive or relapsed non-small cell lung cancer (NSCLC). J Clin Oncol 32, (suppl; abstr 8133) (2014).

[b16] Reck M. . CA184-104: Randomized, multicenter, double-blind, phase III trial comparing the efficacy of ipilimumab (Ipi) with paclitaxel/carboplatin (PC) versus placebo with PC in patients (pts) with stage IV/recurrent non-small cell lung cancer (NSCLC) of squamous histology. J Clin Oncol 31, (suppl; abstr 8117) (2013).

[b17] Brahmer J. . Nivolumab versus Docetaxel in Advanced Squamous-Cell Non-Small-Cell Lung Cancer. N Engl J Med 373, 123–135 (2015). 26028407 10.1056/NEJMoa1504627 PMC4681400

[b18] Forde P. M., Kelly R. J. & Brahmer J. R. New strategies in lung cancer: translating immunotherapy into clinical practice. Clin Cancer Res 20, 1067–1073 (2014). 24470514 10.1158/1078-0432.CCR-13-0731

[b19] Singh B. H. & Gulley J. L. Immunotherapy and therapeutic vaccines in prostate cancer: an update on current strategies and clinical implications. Asian J Androl 16, 364–371 (2014). 24435055 10.4103/1008-682X.122585 PMC4023361

[b20] Digumarti R. . A randomized, double-blind, placebo-controlled, phase II study of oral talactoferrin in combination with carboplatin and paclitaxel in previously untreated locally advanced or metastatic non-small cell lung cancer. J Thorac Oncol 6, 1098–1103 (2011). 21532506 10.1097/JTO.0b013e3182156250

[b21] Parikh P. M. . Randomized, Double-Blind, Placebo-Controlled Phase II Study of Single-Agent Oral Talactoferrin in Patients With Locally Advanced or Metastatic Non-Small-Cell Lung Cancer That Progressed After Chemotherapy. J Clin Oncol 29, 4129–4136 (2011). 21969509 10.1200/JCO.2010.34.4127

[b22] Ramalingam S. . Talactoferrin alfa versus placebo in patients with refractory advanced non-small-cell lung cancer (FORTIS-M trial). Ann Oncol 24, 2875–2880 (2013). 24050956 10.1093/annonc/mdt371

[b23] Olivo S. A. . Scales to assess the quality of randomized controlled trials: a systematic review. Phys Ther 88, 156–175 (2008). 18073267 10.2522/ptj.20070147

[b24] Parmar M. K., Torri V. & Stewart L. Extracting summary statistics to perform meta-analyses of the published literature for survival endpoints. Stat Med 17, 2815–2834 (1998). 9921604 10.1002/(sici)1097-0258(19981230)17:24<2815::aid-sim110>3.0.co;2-8

[b25] Tierney J. F., Stewart L. A., Ghersi D., Burdett S. & Sydes M. R. Practical methods for incorporating summary time-to-event data into meta-analysis. Trials 8, 16 (2007). 17555582 10.1186/1745-6215-8-16 PMC1920534

[b26] Higgins J. P., Thompson S. G., Deeks J. J. & Altman D. G. Measuring inconsistency in meta-analyses. BMJ 327, 557–560 (2003). 12958120 10.1136/bmj.327.7414.557 PMC192859

[b27] Neninger V. E. . Phase II randomized controlled trial of an epidermal growth factor vaccine in advanced non-small-cell lung cancer. J Clin Oncol 26, 1452–1458 (2008). 18349395 10.1200/JCO.2007.11.5980

[b28] Manegold C. . Randomized Phase II Trial of a Toll-Like Receptor 9 Agonist Oligodeoxynucleotide, PF-3512676, in Combination With First-Line Taxane Plus Platinum Chemotherapy for Advanced-Stage Non-Small-Cell Lung Cancer. J Clin Oncol 26, 3979–3986 (2008). 18711188 10.1200/JCO.2007.12.5807

[b29] Butts C. . Updated survival analysis in patients with stage IIIB or IV non-small-cell lung cancer receiving BLP25 liposome vaccine (L-BLP25): phase IIB randomized, multicenter, open-label trial. J Cancer Res Clin 137, 1337–1342 (2011). 10.1007/s00432-011-1003-3 PMC11828286 21744082

[b30] Quoix E. . Therapeutic vaccination with TG4010 and first-line chemotherapy in advanced non-small-cell lung cancer: a controlled phase 2B trial. Lancet Oncol 12, 1125–1133 (2011). 22019520 10.1016/S1470-2045(11)70259-5

[b31] Shtivelband M. . Randomized, blinded, placebo-controlled phase II trial of docetaxel and bavituximab as second-line therapy in locally advanced or metastatic non-squamous non-small cell lung cancer. J Clin Oncol 31, (suppl; abstr 8095) (2013).

[b32] Butts C. . Tecemotide (L-BLP25) versus placebo after chemoradiotherapy for stage III non-small-cell lung cancer (START): a randomised, double-blind, phase 3 trial. Lancet Oncol 15, 59–68 (2014). 24331154 10.1016/S1470-2045(13)70510-2

[b33] Alfonso S. . A Randomized, Multicenter, Placebo-Controlled Clinical Trial of Racotumomab-Alum Vaccine as Switch Maintenance Therapy in Advanced Non-Small Cell Lung Cancer Patients. Clin Cancer Res 20, 3660–3671 (2014). 24788102 10.1158/1078-0432.CCR-13-1674

[b34] Giaccone G. . A phase III study of belagenpumatucel-L, an allogeneic tumour cell vaccine, as maintenance therapy for non-small cell lung cancer. Eur J Cancer 51, 2321–2329 (2015). 26283035 10.1016/j.ejca.2015.07.035

[b35] EA Q. . Results of the phase IIb part of TIME study evaluating TG4010 immunotherapy in stage IV non-small cell lung cancer (NSCLC) patients receiving first line chemotherapy. J Clin Oncol 33, (suppl; abstr 3034) (2015).

[b36] Lynch T. J. . Ipilimumab in Combination With Paclitaxel and Carboplatin As First-Line Treatment in Stage IIIB/IV Non-Small-Cell Lung Cancer: Results From a Randomized, Double-Blind, Multicenter Phase II Study. J Clin Oncol 30, 2046–2054 (2012). 22547592 10.1200/JCO.2011.38.4032

[b37] Borghaei H. . Nivolumab versus Docetaxel in Advanced Nonsquamous Non-Small-Cell Lung Cancer. N Engl J Med 373, 1627–1639 (2015). 26412456 10.1056/NEJMoa1507643 PMC5705936

[b38] Spira A. I. . Efficacy, safety and predictive biomarker results from a randomized phase II study comparing MPDL3280A vs. docetaxel in 2L/3L NSCLC (POPLAR). J Clin Oncol 33, (suppl; abstr 8010) (2015).

[b39] O’Brien M. E. R. SRL172 (killed Mycobacterium vaccae) in addition to standard chemotherapy improves quality of life without affecting survival, in patients with advanced non-small-cell lung cancer: phase III results. Ann Oncol 15, 906–914 (2004). 15151947 10.1093/annonc/mdh220

[b40] Ridolfi L. . Chemotherapy with or without low-dose interleukin-2 in advanced non-small cell lung cancer: results from a phase III randomized multicentric trial. Int J Oncol 39, 1011–1017 (2011). 21720704 10.3892/ijo.2011.1099

[b41] Zhong R., Teng J., Han B. & Zhong H. Dendritic cells combining with cytokine-induced killer cells synergize chemotherapy in patients with late-stage non-small cell lung cancer. Cancer Immunol Immun 60, 1497–1502 (2011). 10.1007/s00262-011-1060-0 PMC11029021 21681372

[b42] Wu C., Jiang J., Shi L. & Xu N. Prospective study of chemotherapy in combination with cytokine-induced killer cells in patients suffering from advanced non-small cell lung cancer. Anticancer Res 28, 3997–4002 (2008). 19192663

[b43] Bradbury P. A. & Shepherd F. A. Immunotherapy for lung cancer. J Thorac Oncol 3, S164–S170 (2008). 18520304 10.1097/JTO.0b013e318174e9a7

[b44] Ruiz R., Hunis B. & Raez L. E. Immunotherapeutic Agents in Non-small-cell Lung Cancer Finally Coming to the Front Lines. Curr Oncol Rep 16, 1–10 (2014). 10.1007/s11912-014-0400-6 25030654

[b45] Nemunaitis J. . Phase II study of belagenpumatucel-L, a transforming growth factor beta-2 antisense gene-modified allogeneic tumor cell vaccine in non-small-cell lung cancer. J Clin Oncol 24, 4721–4730 (2006). 16966690 10.1200/JCO.2005.05.5335

[b46] Crombet Ramos T. . Treatment of NSCLC Patients with an EGF-Based Cancer Vaccine. Cancer Biol Ther 5, 145–149(2006). 16357522 10.4161/cbt.5.2.2334

[b47] Neninger E. . Combining an EGF-based cancer vaccine with chemotherapy in advanced nonsmall cell lung cancer. J Immunother 32, 92–99 (2009). 19307998 10.1097/CJI.0b013e31818fe167

[b48] Pardoll D. M. The blockade of immune checkpoints in cancer immunotherapy. Nat Rev Cancer 12, 252–264 (2012). 22437870 10.1038/nrc3239 PMC4856023

[b49] Helissey C., Champiat S. & Soria J. Immune checkpoint inhibitors in advanced nonsmall cell lung cancer. Curr Opin Oncol 27, 108–117 (2015). 25602683 10.1097/CCO.0000000000000167

[b50] Topalian S. L. . Safety, activity, and immune correlates of anti-PD-1 antibody in cancer. N Engl J Med 366, 2443–2454 (2012). 22658127 10.1056/NEJMoa1200690 PMC3544539

[b51] Marten A. . Interactions between dendritic cells and cytokine-induced killer cells lead to an activation of both populations. J Immunother 24, 502–510 (2001). 11759073 10.1097/00002371-200111000-00007

[b52] Gelao L., Criscitiello C., Esposito A., Goldhirsch A. & Curigliano G. Immune Checkpoint Blockade in Cancer Treatment: A Double-Edged Sword Cross-Targeting the Host as an “Innocent Bystander”. Toxins 6, 914–933 (2014). 24594636 10.3390/toxins6030914 PMC3968368

[b53] Weber J. S., Yang J. C., Atkins M. B. & Disis M. L. Toxicities of Immunotherapy for the Practitioner. J Clin Oncol 33, 2092–2099 (2015). 25918278 10.1200/JCO.2014.60.0379 PMC4881375

[b54] Wang M. . Evaluation of tumour vaccine immunotherapy for the treatment of advanced non-small cell lung cancer: a systematic meta-analysis. BMJ Open 5, e6321 (2015). 10.1136/bmjopen-2014-006321 PMC4401843 25872936

[b55] Wang S. & Wang Z. Efficacy and safety of dendritic cells co-cultured with cytokine-induced killer cells immunotherapy for non-small-cell lung cancer. Int Immunopharmacol 28, 22–28 (2015). 26013109 10.1016/j.intimp.2015.05.021

[b56] Li H. . Dendritic cell-activated cytokine-induced killer cells enhance the anti-tumor effect of chemotherapy on non-small cell lung cancer in patients after surgery. Cytotherapy 11, 1076–1083 (2009). 19929470 10.3109/14653240903121252

[b57] Zhao M., Li H., Li L. & Zhang Y. Effects of a gemcitabine plus platinum regimen combined with a dendritic cell-cytokine induced killer immunotherapy on recurrence and survival rate of non-small cell lung cancer patients. Exp Ther Med 7, 1403–1407 (2014). 24940447 10.3892/etm.2014.1574 PMC3991503

[b58] Shi S. B. . Efficacy of erlotinib plus dendritic cells and cytokine-induced killer cells in maintenance therapy of advanced non-small cell lung cancer. J Immunother 37, 250–255 (2014). 24714359 10.1097/CJI.0000000000000015

[b59] Shi S. B., Ma T. H., Li C. H. & Tang X. Y. Effect of maintenance therapy with dendritic cells: cytokine-induced killer cells in patients with advanced non-small cell lung cancer. Tumori 98, 314–319 (2012). 22825506 10.1177/030089161209800306

[b60] Yang L. . Enhanced antitumor effects of DC-activated CIKs to chemotherapy treatment in a single cohort of advanced non-small-cell lung cancer patients. Cancer Immunol Immun 62, 65–73 (2013). 10.1007/s00262-012-1311-8 PMC11028994 22744010

