# Supplementary material for: The efficacy and safety of immunotherapy in patients with advanced NSCLC: a systematic review and meta-analysis
Source: Sci Rep. 2016 Aug 25;6:32020. doi: 10.1038/srep32020 (PMC4997317; doi:10.1038/srep32020)
Supplement: Supplementary Information [file srep32020-s1.doc]

**The efficacy and safety of immunotherapy in patients with advanced NSCLC: a systematic review and meta-analysis**

**Liang Zhou**^1, +^**, Xi-Ling Wang**^1, +^**, Qing-Long Deng**^1^**, Yan-Qiu Du**^2^ **and Nai-Qing Zhao^1, *^**

^1^ Department of Biostatistics, School of Public Health and Key Laboratory of Public Health Safety, Fudan University, Shanghai 200032, People’s Republic of China

^2^ Department of Preventive Medicine, Fudan University, Shanghai 200032, People’s Republic of China

*Email: [nqzhao@fudan.edu.cn](mailto:nqzhao@fudan.edu.cn)

**Search Strategy**

1. exp Lung Neoplasms/
2. exp Carcinoma, Non-Small-Cell Lung/
3. nsclc.tw.
4. (lung$ or pulmonary or bronchus or bronchogenic or bronchial or bronchoalveolar or alveolar).tw.
5. (non small cell or non-oat cell).tw.
6. (cancer or carcinoma$ or neoplasm$ or malignan$ or tumo?r).tw.
7. 4 and 5 and 6
8. 1 or 2 or 3 or 7
9. exp immunotherapy/
10. exp cancer vaccine/
11. exp cell cycle checkpoints/
12. exp cytokines/tu
13. exp Immunologic Factors/tu
14. exp dendritic cells/
15. exp cytokine-induced killer cells/
16. (GVAX or Tecetomide or L-BLP25 or MAGE-A3 or EGF or Belagenpumatucel-L or Tergenpumatucel-L or TG4010 or CIMAvax or Racotumomab).tw.
17. (CTLA-4 or Ipilimumab or tremelimumab or PD-1 or nivolumab or pembrolizumab or MK-3475 or PD-L1 or Nivolumab or BMS-936559 or MPDL3280A).tw.
18. (IL-2 or DC or CIK or Talactoferrin or TLF).tw.
19. 9 or 10 or 11 or 12 or 13 or 14 or 15 or 16 or 17 or 18
20. 8 and 19
21. limit 20 to human
22. limit 21 to (controlled clinical trial or randomized controlled trial)
23. limit 22 to yr="2003 -Current"
24. limit 23 to english language
